# Immunogenicity of a Candidate Live Attenuated Vaccine for Rift Valley Fever Virus with a Two-Segmented Genome

**DOI:** 10.1089/vim.2022.0104

**Published:** 2023-01-16

**Authors:** Victoria B. Ayers, Yan-Jang S. Huang, James I. Dunlop, Alain Kohl, Benjamin Brennan, Stephen Higgs, Dana L. Vanlandingham

**Affiliations:** ^1^ Department of Diagnostic Medicine/Pathobiology, College of Veterinary Medicine, Kansas State University, Manhattan, Kansas, USA.; ^2^ Biosecurity Research Institute, Kansas State University, Manhattan, Kansas, USA.; ^3^ MRC-University of Glasgow Centre for Virus Research, Glasgow, United Kingdom.

**Keywords:** Rift Valley fever virus, RVFV, MP12, live attenuated vaccine, double deletion

## Abstract

Rift Valley fever virus (RVFV) is an emerging arbovirus that affects both ruminants and humans. RVFV causes severe and recurrent outbreaks in Africa and the Arabian Peninsula with a significant risk for emergence into new locations. Although there are a variety of RVFV veterinary vaccines for use in endemic areas, there is currently no licensed vaccine for human use; therefore, there is a need to develop and assess new vaccines. Herein, we report a live-attenuated recombinant vaccine candidate for RVFV, based on the previously described genomic reconfiguration of the conditionally licensed MP12 vaccine. There are two general strategies used to develop live-attenuated RVFV vaccines, one being serial passage of wild-type RVFV strains to select attenuated mutants such as Smithburn, Clone 13, and MP12 vaccine strains. The second strategy has utilized reverse genetics to attenuate RVFV strains by introducing deletions or insertions within the viral genome. The novel candidate vaccine characterized in this report contains a two-segmented genome that lacks the medium viral segment (M) and two virulence genes (nonstructural small and nonstructural medium). The vaccine candidate, named r2segMP12, was evaluated for the production of neutralizing antibodies to RVFV in outbred CD-1 mice. The immune response induced by the r2segMP12 vaccine candidate was directly compared to the immune response induced by the rMP12 parental strain vaccine. Our study demonstrated that a single immunization with the r2segMP12 vaccine candidate at 10^5^ plaque-forming units elicited a higher neutralizing antibody response than the rMP12 vaccine at the same vaccination titer without the need for a booster.

## Introduction

Rift Valley fever virus (RVFV; *Phenuiviridae*, *Phlebovirus*) is a clinically important mosquito-borne pathogen causing disease in both humans and ruminants. Although most humans have no clinical sign, others develop flu-like symptoms with headaches, fever, or myalgia (Hartman, 2017; Laughlin et al, 1979; Wichgers Schreur et al, 2020), and around 1% of infections can progress to life-threatening diseases, including encephalitis, hemorrhagic fever, or thrombosis (Ikegami and Makino, 2011). While humans are considered dead-end hosts for RVFV (Chevalier et al, 2010), ruminants, especially sheep and goats, act as amplifying hosts (Hartman, 2017). In livestock, death from the disease is most commonly caused by abortion storms with abortion rates of up to 100% (Hartman, 2017; Wichgers Schreur et al, 2021). Other clinical signs in livestock for RVFV include hyperthermia, nasal and ocular secretions, and/or abdominal colic (Ikegami, 2017; Kwasnik et al, 2021).

The epidemiology of RVFV is multifactorial involving complex relationships and dynamics between ruminants, humans, and mosquitoes (Hartman, 2017). Transmission to livestock and humans usually occurs through the bite of an infected mosquito or by direct contact with infected tissues, blood, or bodily fluids of infected animals. Infections may also result by exposure to the virus through aerosolization (Kwasnik et al, 2021; Pepin et al, 2010). Although the virus is endemic in sub-Saharan Africa and the Arabian Peninsula, susceptible ruminants and mosquito vectors are found in many nonendemic countries.

Sheep, goats, and cattle are the main ruminants that pose a risk of causing RVFV outbreaks, especially if involved in importation from endemic countries (Abdo-Salem et al, 2011; Chevalier et al, 2010; Shoemaker et al, 2002). In addition to livestock, mosquito vectors, mainly of the *Aedes* and *Culex* genera, increase the likelihood of RVFV dispersal and establishment in nonendemic regions (Javelle et al, 2020).

Vaccination is the most effective method for preventing and controlling RVFV outbreaks (Ikegami and Makino, 2009). Currently, there is no licensed vaccine or antiviral treatment for humans or animals in nonendemic countries (Faburay et al, 2017). Multiple veterinary vaccines are available and commonly used in livestock in endemic counties, including the Smithburn strain and the Clone 13 strain (Alhaj, 2016). The Smithburn strain was developed in 1971 by serial intracerebral passage in mice (Alhaj, 2016; Smithburn, 1949). Although the Smithburn vaccine is immunogenic, it exhibits a partially attenuated phenotype and cannot be used for the immunization of young and pregnant ruminants (Botros et al, 2006; Coetzer and Barnard, 1977; Ikegami and Makino, 2009; Kamal, 2009).

The Clone 13 strain is a naturally attenuated RVFV strain that contains a deletion in the nonstructural small (NSs) gene (Muller et al, 1995). The Clone 13 strain vaccine has not only been shown to be safe and effective in lambs, cattle, and pregnant ewes (Dungu et al., 2010; Makoschey et al, 2016) but is also partially attenuated, as observed with vertical transmission and teratogenic effects in ewes after the administration of high doses (Makoschey et al, 2016). The MP12 vaccine is conditionally licensed for use in ruminants in the United States (Ikegami et al, 2015; Miller et al, 2015), and was produced through serial passage of the ZH548 RVFV strain in the presence of 5-flurouracil (Caplen et al, 1985). The virus was found to be attenuated and protective in mice, lambs, and calves (Caplen et al, 1985; Wilson et al, 2014).

Several approaches have been taken to address the limitations of the available candidate RVFV vaccines (Billecocq et al, 2008; Dunlop et al, 2018; Habjan et al, 2008; Ikegami et al, 2006) through the gene deletions of one or both virulence factors (NSs and non-structural medium [NSm]). NSs is a nonstructural protein that facilitates evasion of the host innate immune system (Brennan et al, 2014), while the NSm nonstructural protein promotes suppression of apoptosis in infected hosts (Ikegami and Makino, 2011). More importantly, the deletion of NSm has previously resulted in the reduced ability of RVFV to infect, replicate, and disseminate from the midgut epithelial cells in *Aedes* mosquitoes (Kading et al, 2014).

Brennan et al developed a candidate vaccine based on the attenuated MP12 strain that lacks the NSs and NSm genes in a reconfigured two-segmented genome, designated r2segMP12 (Brennan et al, 2011). The rationally designed recombinant r2segMP12 strain aims to further enhance the safety profile of the MP12 strain based on previously published work (Bird et al, 2008; Ikegami et al, 2006; Won et al, 2007; Won et al, 2006).

In this study, the immunogenicity of the r2segMP12 vaccine candidate was evaluated by quantifying the serum neutralizing activity in CD-1 mice. Groups of mice were subcutaneously inoculated with different titers of the vaccine candidate on day 0, followed by a booster dose on day 21 after initial immunization. Serum samples were collected at 20 and 42 days after initial immunization and evaluated using plaque reduction neutralization tests (PRNT) for a neutralizing antibody titer at or above the threshold antibody level for protection.

The neutralizing antibody response produced by the different titers of r2segMP12 was compared between the use of a single dose versus a single dose followed by a booster dose. Neutralizing antibodies produced following the r2segMP12 vaccine and the rMP12 parental strain vaccine were also examined. Taken together, these data demonstrated that the double deletion of NSs and NSm genes does not reduce the immunogenicity of the MP12 vaccine strain.

## Materials and Methods

### Cell lines and viruses

**FIG. 1.**
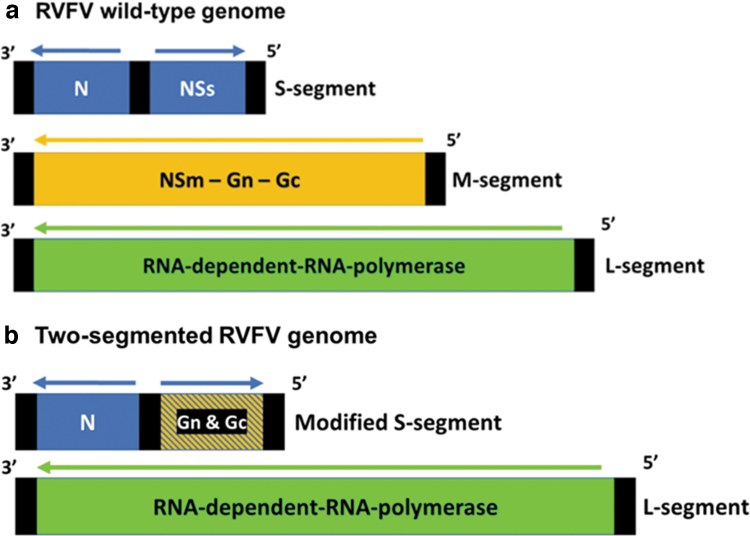
Schematic comparing the RVFV genome with the modified recombinant two-segmented RVFV genome. **(a)** The RVFV wild-type genome, which includes the ambisense small (S-) segment consisting of the nucleocapsid (N) protein and the nonstructural protein, NSs; the negative-sense medium (M-) segment, which contains the structural proteins, Gn and Gc, and the nonstructural proteins, NSm and 78kD; and the negative-sense large (L-) segment containing the L protein or RNA-dependent-RNA-polymerase. **(b)** The modified recombinant bisegmented RVFV genome, which only contains the S-segment and the L-segment. The NSs coding sequence has been replaced with the Gn and Gc precursors, maintaining the ambisense coding strategy, and the genome is lacking the authentic M RNA segment. NSm, nonstructural medium; NSs, nonstructural small; RVFV, Rift Valley fever virus.

The r2segMP12 recombinant vaccine candidate for RVFV was produced in a previously published study as summarized in Figure 1 (Brennan et al, 2011). The vaccine candidate was propagated and titered in African green monkey kidney epithelial (Vero76) cells maintained at 37°C in Leibovitz's L-15 media (Thermo Fisher Scientific, Waltham, MA) supplemented with 10% fetal bovine serum (Thermo Fisher Scientific), 10% tryptose phosphate broth (Sigma-Aldrich, St. Louis, MO), penicillin/streptomycin (Thermo Fisher Scientific), and L-glutamine (Thermo Fisher Scientific) as previously described (Ayers et al, 2018). The Vero76 cell line was obtained from the collection of Instituto Conmemorativo Gorgas de Estudios de la Salud. The rMP12 parental strain vaccine was generated using a control rescue experiment as previously described (Brennan et al, 2011); and was then propagated and titered in Vero76 cells for use as a positive control (Caplen et al, 1985). Viral titers were determined using plaque assay as previously described (Nuckols et al, 2015).

### Animals and immunizations

The following experimental procedures and handling of live animals were approved by the Kansas State University Institutional Animal Care and Use Committee. All methods were carried out in accordance with the approved protocol and relevant regulations. To determine the immunization regimens required to elicit protective neutralizing antibody responses for the r2segMP12 vaccine candidate, fifty 3–4-week-old, outbred CD-1 mice (Charles River, Raleigh, NC) were subcutaneously immunized with one of the following: r2segMP12 vaccine candidate, rMP12 parental strain vaccine, or sterile L-15 media. Animals were randomly assigned into 10 groups of 5, representing 8 experimental regimens, using increasing doses (10^3^, 10^4^, or 10^5^ plaque-forming unit [PFU]) of infectious viruses in a single immunization or two immunizations (Table 1).

**Table 1.** Immunization Regimen of CD-1 Mice (Dosages Are Calculated in PFU/Mouse)

Group	Number of mice	Dosage of 1st immunization (Day 0)	Dosage of 2nd immunization (Day 21)
1	5	10^3^ (r2segMP12)	No injection
2	5	10^4^ (r2segMP12)	No injection
3	5	10^5^ (r2segMP12)	No injection
4	5	0 (sterile media; mock control)	No injection
5	5	10^5^ (parental strain rMP12 control)	No injection
6	5	10^3^ (r2segMP12)	10^5^ (r2segMP12)
7	5	10^4^ (r2segMP12)	10^5^ (r2segMP12)
8	5	10^5^ (r2segMP12)	10^5^ (r2segMP12)
9	5	Sterile media; mock control	Sterile media; mock control
10	5	10^5^ (parental rMP12 strain control)	10^5^ (parental rMP12 strain control)

PFU, plaque-forming unit.

The regimen of a single immunization administered at an increasing dosage per group (*n* = 5) was included to determine the correlation of neutralizing antibodies produced by different dosages of the r2segMP12 strain. Three groups of mice (*n* = 5) received a second immunization of the r2segMP12 strain to evaluate the effect of a booster immunization using the dose of the vaccine that produced the highest neutralizing antibody titer after the primary vaccine (10^5^ PFU). In addition to the experimental groups that received the r2segMP12 vaccine candidate, four additional groups (*n* = 5) were designated as control groups, with two positive control groups receiving the rMP12 vaccine at 10^5^ PFU and two negative control groups receiving an equal volume of sterile culture L-15 media.

Mice were immunized subcutaneously on day 0 of the experiment with an equal volume of their corresponding immunization (r2segMP12, rMP12, and L-15 media). Before the initial immunization, all mice were determined to be healthy and seronegative to RVFV through the analysis of collected serum using PRNT (data not shown). Animals were maintained for 6 weeks after the initial immunization. Mice were immobilized using an isoflurane vaporizer before blood collection. Whole blood samples of 0.1 mL were collected from the lateral saphenous vein from immunized animals at 20 days after initial immunization using a 22 g needle. Serum samples were obtained through centrifugation of coagulated blood at 2,000 *g* for 10 min at 4°C and used for the detection of neutralizing antibodies using PRNT. Terminal bleeds were collected at 42 days after initial immunization by cardiac puncture following isoflurane anesthesia and death was confirmed by cervical dislocation.

### Plaque reduction neutralization test

PRNT were used to determine which vaccination regimen produced the highest titer of neutralizing antibodies following the protocol previously described (Roehrig et al, 2008). All serum samples were inactivated at 56°C for 30 min and dilutions between 1:10 and 1:320 were tested (Roehrig et al, 2008). Approximately 50 PFU of the rMP12 vaccine strain was added to each serum concentration and incubated for 1 h at 37°C before infecting the Vero76 cells in 24-well plates. The wells were then washed with Dulbecco's phosphate-buffered saline and overlaid with 1% methylcellulose.

After 5 days of incubation at 37°C, plaques were counted, and the neutralizing antibody titers were calculated based on a 50% or greater reduction in plaques from the positive control (PRNT_50_). Seroconversion was defined using the cutoff of 1:10 PRNT_50_ titer, a seropositive threshold commonly used for assessing the neutralizing antibody responses elicited by arbovirus vaccines (Julander et al, 2011; Roehrig et al, 2008; Van Gessel et al, 2011).

### Statistical analysis

The PRNT_50_ titers of animals receiving each dosage of the vaccine candidate were compared at 20 days using a Kruskal–Wallis test followed by Dunnett's test as the *post hoc* multiple comparison procedure, including a comparison of each dosage to the parental strain rMP12-positive control.

Using a Kruskal–Wallis test followed by a Dunnett's test *post hoc*, PRNT_50_ titers of animals receiving a single dose of the r2segMP12 vaccine candidate at varying titers were compared to mice receiving both an initial dose of the r2segMP12 vaccine candidate at varying titers and a booster vaccine at 10^5^ PFU at 42 days postimmunization (dpi). Finally, PRNT_50_ titers of animals receiving only a single dosage of the r2segMP12 vaccine candidate at 10^5^ PFU were compared to animals receiving the rMP12 vaccine at 10^5^ PFU at 42 dpi with a Mann–Whitney test. All tests were performed using the GraphPad Prism (version 8.1.2) program (GraphPad Software, Inc., San Diego, CA).

## Results

**FIG. 2.**
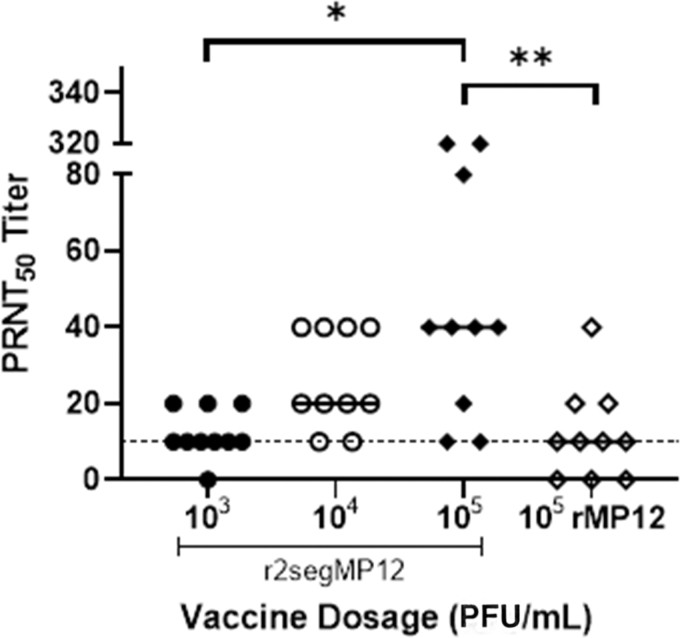
Comparison of r2segMP12 and rMP12 neutralizing antibody response. CD-1 mice (*n* = 10 per group) were administered either r2segMP12 stain at one of three titers (10^3^, 10^4^, or 10^5^ PFU) or rMP12 strain (10^5^ PFU). Serum was collected at 20 dpi and antibody titer was measured by PRNT_50_ with a 1:10 neutralizing antibody titer used as the threshold for the correlate of protection (*dotted line*). The Kruskal–Wallis test with Dunnett's *post hoc* multiple comparison test was used. “*” Indicates the significant difference identified by the Kruskal–Wallis test plus Dunnett's *post hoc* test (*p* = 0.0151). “**” Indicated the significant difference identified by the Kruskal–Wallis test plus Dunnett's *post hoc* test (*p* = 0.0087). The bar lines represent the medians of values from that group of animals. PFU, plaque-forming units; PRNT, plaque reduction neutralization tests.

Animals from all groups did not show any adverse clinical sign during the experiment. All animals were bled at 20 dpi to evaluate if one single immunization with the r2segMP12 vaccine candidate elicits neutralizing antibody responses above the threshold for the correlate of protection (PRNT_50_ > 10). All, but one mouse immunized with the r2segMP12 vaccine candidate seroconverted after a single immunization (Fig. 2).

Therefore, the r2segMP12 vaccine candidate was capable of eliciting neutralizing antibody responses in CD-1 mice at dosages between 10^3^ and 10^5^ PFU. Mice that received a single immunization of the r2segMP12 vaccine candidate at 10^5^ PFU produced a significantly higher number of neutralizing antibodies than mice that received a single immunization of the r2segMP12 vaccine candidate at 10^3^ PFU (Fig. 2, *p* = 0.0139), demonstrating a dose–response relationship in the vaccine immunogenicity. Importantly, the comparison of immunogenicity with the rMP12 vaccine strain suggests the superior immunogenicity of the r2segMP12 strain. Mice immunized with r2segMP12 at a titer of 10^5^ PFU had a significantly higher neutralizing antibody response compared to mice that received the rMP12 vaccine at the same titer (Fig. 2, *p* = 0.0079).

**FIG. 3.**
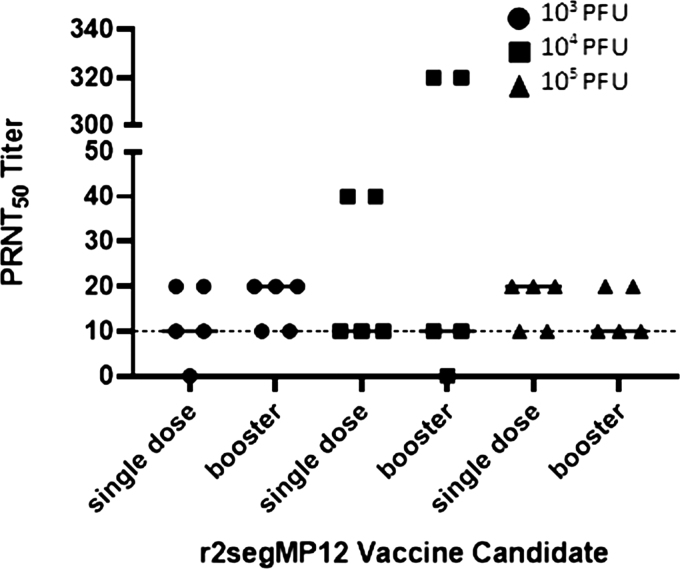
Comparison of PRNT_50_ titers from different doses of the r2segMP12 vaccine candidate on 42dpi. CD-1 mice (*n* = 5 per group) were administered either a single dose of r2segMP12 (at either 10^3^ [*filled circles*], 10^4^ [*filled squares*], or 10^5^ [*filled triangles*] PFU), or two doses (single dose and a booster dose) at the same titer (primary immunization at either 10^3^, 10^4^, or 10^5^ PFU; booster at 10^5^ PFU). Serum neutralizing activity was measured by PRNT_50_ and compared by Kruskal–Wallis test followed by a Dunnett's test as the *post hoc* multiple comparison procedure. The bars represent the median and the threshold of protection is marked by the *dotted line*.

To determine if a booster immunization of the r2segMP12 strain can increase immunogenicity and produce long-lasting neutralizing antibody responses, the PRNT_50_ titers were measured in mice that received varying initial titers of the r2segMP12, followed by a booster at 21 dpi. Animals in groups 6, 7, and 8 (Table 1) received a booster of the r2segMP12 vaccine candidate at a titer of 10^5^ PFU at 21 dpi (Fig. 3).

There was no significant difference in the PRNT_50_ titers between mice that received one single immunization of the r2segMP12 strain and mice that received a final boost at 21 dpi. While two mice that received the r2segMP12 vaccine at 10^4^ PFU with the addition of the booster had a slightly higher neutralizing antibody response than mice that received the 10^5^ PFU vaccine and booster, there was no significant difference in the group as a whole. These results demonstrated the immunogenicity of the r2segMP12 strain, with neutralizing antibody titers suggestive of protection.

**FIG. 4.**
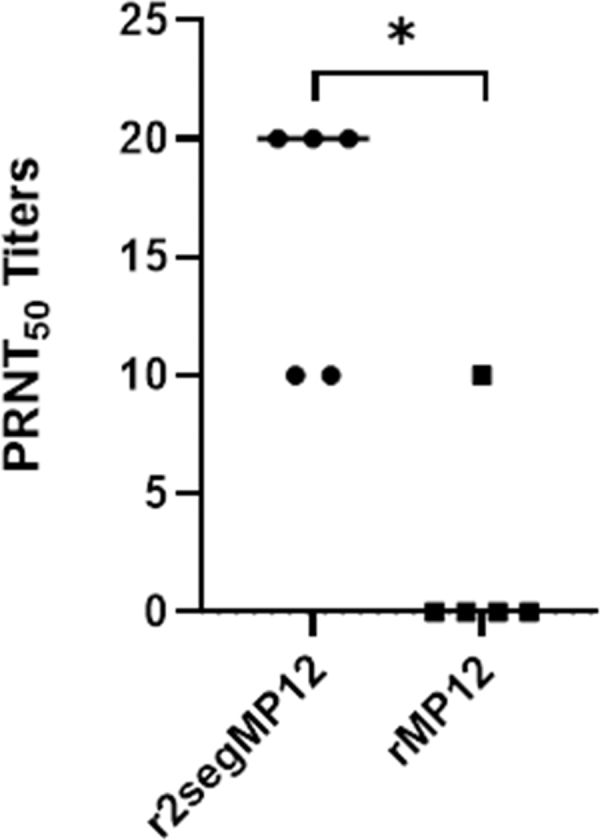
Comparison of PRNT_50_ titers between mice receiving one single immunization with the rMP12 strain and the r2segMP12 strain at 42 days after immunization. CD-1 mice (*n* = 5 per group) were administered a single dose of either r2segMP12 or rMP12 at 10^5^ PFU/mL and antibody titer was measured by PRNT_50_ and compared by Mann–Whitney test with the bars representing the median. *Indicates *p* < 0.05.

Given the observation that a single dose of the r2segMP12 vaccine candidate at 10^5^ PFU elicited a serum neutralizing antibody response at 42 dpi, the level of antibody production was next compared with the neutralizing antibody response induced by a single dose of the rMP12 vaccine administered at the same titer. Intriguingly, a single dose of the r2segMP12 strain produced a significantly higher titer of neutralizing antibodies than the rMP12 vaccine (Fig. 4, *p = 0.0238). In addition, serum neutralizing titers in four out of five mice immunized with the rMP12 vaccine wane below PRNT_50_ titer of 10, demonstrating the need for a booster immunization. These data suggest that the r2segMP12 strain is superior to the rMP12 vaccine in eliciting neutralizing antibody responses in mice.

Collectively, these data suggest that the r2segMP12 strain is immunogenic and can elicit neutralizing antibody responses in CD-1 mice that received one single immunization. In addition, the lack of the NSs and NSm genes ensures the safety, but does not compromise the immunogenicity of the r2segMP12 vaccine strain.

## Discussion

Due to the impact of RVFV on both human and livestock health, efforts to prevent and control RVFV have been continuous, however, the limitations of each vaccine, multiple doses required, and expenses to maintain these regimens have made it difficult (Mackenzie, 1935; Pittman et al, 1999; Randall et al, 1962). While live attenuated vaccines have been developed for RVFV in an effort to eliminate the need for booster inoculations, several vaccines have demonstrated to be partially attenuated and cause teratogenic effects and abortions in livestock (Hunter et al, 2002; Morrill et al, 1997). These disadvantages are problematic, especially in nonendemic areas during epidemic periods.

There is an urgent need to develop a new vaccine against RVFV. Therefore, this study sought to establish the immunogenicity of a recombinant RVFV vaccine, containing a two-segmented viral genome in outbred CD-1 mice. Altogether, the observations made demonstrated that a single dose of the r2segMP12 strain induced a neutralizing antibody response in mice. The neutralizing antibody responses identified in this study should protect following a challenge with RVFV, although previous studies have shown certain recombinant vaccines to be protective against lethal RVFV strains with neutralizing antibody titers as low as 1:4 (Wallace et al, 2006).

The candidate vaccine used in this study was rationally designed through the deletion of virulence factors, NSs and NSm, which is based on previous work and because they have been shown to be virulence factors for wild-type RVFV (Bird et al, 2011; Ikegami et al, 2006; Won et al, 2007). There is also evidence of the NSs protein contributing to RVFV disease outcome in mice by modulating host cell features and defense mechanisms (Leger et al, 2020). Previously, the generation of viruses lacking the NSs gene established the product is not essential for replication in mice, making NSs an accessory protein (Bridgen et al, 2001).

In comparison, the Clone 13 vaccine demonstrated to be avirulent in mice and highly immunogenic (Muller et al, 1995). However, even with the deletion of the NSs segment in the Clone 13 strain, it has been reported to cause stillbirths and fetal infections when administered in an overdose to pregnant ewes in their first trimester (Makoschey et al, 2016).

While other RVFV candidate live attenuated vaccines have been developed through the deliberate deletion of NSs and NSm genes and demonstrated to be safe and immunogenic in mice and pregnant sheep (Bird et al, 2011; Bird et al, 2008), our work has important implications for the development of RVFV candidate live attenuated vaccines. While these vaccines were made using similar methods, the r2segMP12 strain with a two-segmented genome will have a reduced likelihood for reversion to the virulent phenotype. In addition, the r2segMP12 strain proves that the double deletion of NSs and NSm genes does not cause reduced immunogenicity relative to the rMP12 strain (Brennan et al, 2011).

The results of this study also determined that the r2segMP12 strain elicited a significantly higher level of neutralizing antibody response than the conditionally licensed rMP12 vaccine at 20 and 42 dpi. In addition, the r2segMP12 strain does not express the NSs and NSm proteins, providing the basis for differentiating infected from vaccinated animals (DIVA) (McElroy et al, 2009). Specifically, the lack of anti-NSs antibodies has been developed as a DIVA marker for serological diagnosis. Hence, the r2segMP12 strain has the potential utility for the deployment of rapid outbreak responses.

We conclude that the superior immunogenicity of the r2segMP12 strain warrants its advancement in the process of vaccine development, including challenge protection studies conducted in mice and then sheep, which are the amplifying hosts for RVFV. Future experiments will focus on the characterization of the immune response induced by r2segMP12 and its ability to protect against a lethal RVFV challenge.
